# Distinct biomarkers for different bones in osteoporosis with rheumatoid arthritis

**DOI:** 10.1186/s13075-019-1956-1

**Published:** 2019-07-15

**Authors:** T. Tomizawa, H. Ito, K. Murata, M. Hashimoto, M. Tanaka, K. Murakami, K. Nishitani, M. Azukizawa, A. Okahata, K. Doi, M. Saito, M. Furu, M. Hamaguchi, T. Mimori, S. Matsuda

**Affiliations:** 1 Department of Orthopaedic Surgery, Kyoto University Graduate School of Medicine, 54 Kawahara-Cho, Shogoin, Sakyo, Kyoto, 606-8507 Japan; 2 Department of Advanced Medicine for Rheumatic Diseases, Kyoto University Graduate School of Medicine, Kyoto, Japan; 3 Department of Rheumatology and Clinical Immunology, Kyoto University Graduate School of Medicine, Kyoto, Japan; 4 Department of Endocrinology and Metabolism, Kyoto Prefectural University of Medicine, Kyoto, Japan

**Keywords:** Bone mineral density, Osteoporosis, Rheumatoid arthritis, Bone metabolism marker, Cohort study

## Abstract

**Background:**

Rheumatoid arthritis (RA) is known to cause secondary osteoporosis and fragility fractures. This study aimed to identify biomarkers predictive of bone mineral density (BMD) change at three anatomical sites in patients with RA.

**Methods:**

We conducted a prospective longitudinal study in patients with RA. In 2012, we recruited 379 patients from an RA cohort, 329 of whom underwent evaluation of blood and urine biomarkers together with measurement of BMD in the lumbar spine, proximal femur, and distal forearm. The BMD in these three regions was reassessed in 2014. We performed multivariate linear regression analysis to identify those factors associated with BMD change.

**Results:**

The averages of age, body mass index, and disease activity score in 28 joints (DAS28) at baseline were 63.2 (minimum to maximum, 32–85), 21.3 (12.3–30.0), and 3.2 (0.1–5.9), respectively. Univariate analysis showed that the annual BMD change was significantly associated with the use of steroid, bisphosphonate (BP) or vitamin D (VitD), and serum homocysteine in the lumber spine; DAS28, the use of BP or VitD, CRP, and anti-cyclic citrullinated peptide antibody (ACPA) in the proximal femur; and the dosage of MTX, the use of BP or VitD, and serum tartrate-resistant acid phosphatase 5b (TRACP-5b) in the distal forearm, respectively.

**Conclusions:**

Predictive biomarkers for BMD change in RA patients differ at each anatomical site. Practitioners should treat each anatomical site with different markers and prescribe osteoporosis drugs to prevent fractures for RA patients.

## Mini abstract

The identity of biomarkers that reliably predict changes of bone mineral density in osteoporosis is controversial. We found that distinct biomarkers are effective to predict the changes of bone mineral density in the lumbar spine, the proximal femur, and the distal forearm in patients with rheumatoid arthritis.

## Introduction

Rheumatoid arthritis (RA) is a predominantly inflammatory arthritis and a well-known cause of secondary osteoporosis. Osteoporosis increases the possibility of fragility fractures of spine, hip, and other sites, which reduce life expectancy compared with that of the nonfracture general population [1]. Even distal forearm fractures result in poor quality of life and impaired activities of daily living and may be associated with high mortality [2]. Osteoporosis secondary to RA has a higher likelihood of fractures compared with primary osteoporosis [3], and this unfortunately has not decreased even after introduction of treat-to-target (T2T) strategies [4].

Osteoporosis increases the risk of fractures at various sites of the body. Jin et al. systematically reviewed the site-specific incidence rates of vertebral, proximal hip, and forearm fractures in patients with RA and reported that age, sex, steroid treatment, RA disease activity, history of fractures, and bisphosphonate use were overall predictors of fracture [5]. However, that study did not report site-specific risk factors. In contrast, Wilson et al. demonstrated significant differences between the mean lumbar or femoral *T*-scores and the radial *T*-scores in osteoporosis patients [6]. Moreover, previous large cohort studies have reported that the risk factors for fractures are similar but distinct for different anatomical sites in patients with RA [7–10]. To introduce preventive measures against these fractures, it is critical to identify any site-specific biomarkers, but these remain to be investigated.

Bone metabolism markers have been studied for many years and have been used for the assessment of fracture risk and to select treatment. Of the known biomarkers, tartrate-resistant acid phosphatase 5b (TRACP-5b), undercarboxylated osteocalcin (Uc-OC), and bone-specific alkaline phosphatase (BAP) have been shown to be useful for bone density, while homocysteine and pentosidine are reported to be excellent specific biomarkers for bone quality [11] and have been demonstrated to be useful for predicting bone mineral density (BMD) and fractures. Moreover, several reports showed that several parameters specific for RA such as rheumatoid factor (RF), anti-cyclic citrullinated peptide (ACPA), Health Assessment Questionnaire (HAQ), steroid use, methotrexate, and bDMARDs are associated with BMD in RA patients [12–15]. However, the markers that are the best predictors for use in BMD management of specific anatomical sites in RA remain controversial, and it is unknown whether these biomarkers are still useful if RA-related demographic data are fully taken into count.

To identify effective biomarkers of changes in BMD at different sites in RA patients, we conducted a longitudinal cohort study. We hypothesized that unique biomarkers existed for different anatomical sites in RA patients.

## Methods

### Study plan and participants

**Fig. 1:**
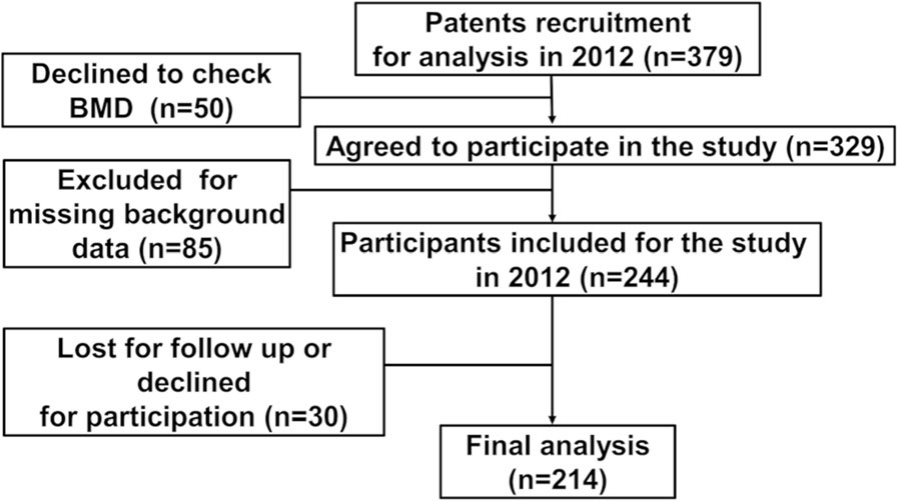
Flow chart of this study

We used the Kyoto University Rheumatoid Arthritis Management Alliance (KURAMA) cohort, which was initiated in May 2011 for prospective monitoring of the changes in condition of patients with RA, with the data to be prospectively used for clinical research [16, 17]. In 2012, we recruited patients specifically for this study and, after obtaining their written informed consent, measured their BMD by dual-energy X-ray absorptiometry (Discovery DXA system, Hologic, Inc) at the lumbar spine, the proximal femur, and the distal forearm. They also underwent clinical examination, serum testing, and urinalysis. These same patients were followed up in 2014, and the same set of data was collected (Fig. 1). The inclusion criteria for this study were as follows: patients who satisfied the 1987 or 2010 American College of Rheumatology/European League Against Rheumatism classification criteria for RA, who had given written informed consent, were over 18 years of age, and for whom a complete set of data was available. The exclusion criteria were as follows: patients who did not agree to participate in the survey or who were lost to follow-up in 2014 or from whom data such as BMD and laboratory data were not obtained. The study was conducted according to the principles of the Declaration of Helsinki and was approved by the Ethics Committee of Kyoto University before the start of the study (E1308).

### Parameter collection

#### Clinical assessments

Clinical parameters of the participants at enrolment include age, sex, female over 60 years old as menopause patients, body mass index (BMI), and Hemoglobin A_1C_ National Glycohemoglobin Standardization Program (HbA1c). Disease activity was recorded as the disease activity score in 28 joints-erythrocyte sedimentation rate (DAS28-ESR) and physical function using a health assessment questionnaire disability index (HAQ). Joint distraction was evaluated with the modified total Sharp score by van der Heijde method (mTSS) [18, 19]. In addition, the use and the dosage of medications for RA such as biological disease-modifying anti-rheumatic drugs (bDMARDs), steroids, methotrexate (MTX), observation period, serum anti-cyclic citrullinated peptide antibody (ACPA), serum rheumatoid factor (RF), and serum C-reactive protein (CRP) were noted and recorded by the attending rheumatologists. The types of medications for osteoporosis were also recorded. CRP was quantified using a latex immunoturbidimetric method. RF was measured by a turbidimetric immunoassay method. ACPA was measured by CLEIA.

#### BMD quantitation

In 2012 and 2014, BMDs in the total lumbar spine, the proximal femur, and the distal forearm were measured as previously described [20, 21]. All data were acquired with the same equipment (Discovery; Hologic, Waltham, MA, USA). BMDs were described using young adult mean (YAM) values. The ROI for the lumbar spine was L2–L4. The ROI for the proximal femur was the femoral neck to the trochanter. The ROI for the distal forearm was the distal one third of the radius and ulna [22–24]. To minimize the effects of the time differences between participants for the two measurements, the annual rate of change of BMD was used as the objective variable, as previously reported [25]. The value was calculated using the formula:

Annual BMD rate of change = (BMD in 2014 − BMD in 2012)/observation period (%/years).

#### Bone metabolism markers

Plasma analysis included tests for homocysteine, TRACP-5b, Uc-OC, and BAP; urine pentosidine was also assayed. Blood was collected in the morning after an overnight fast to minimize daily variation [26]. Homocysteine was measured by high-performance liquid chromatography. TRACP-5b was quantified by enzyme immunoassay. Uc-OC was quantified by electrochemiluminescence immunoassay. BAP was measured by chemiluminescent enzyme immunoassay (CLEIA). Pentosidine was quantified by high-performance liquid chromatography (LSI Medience Corp., Tokyo, Japan).

### Statistics

The data were analyzed using JMP Pro, version 13.0 (SAS, Institute Inc., Cary, NC, USA). Data for continuous variables are expressed as mean and SD (range), and data for categorical variables are expressed as numbers and percentages. For all analyses, we set the annual BMD rate of change for each anatomical site as the objective variable. For univariate analysis, we used simple regression analysis for continuous explanatory variables and Student *t* test for binary explanatory variables. The candidate explanatory parameters of BMD were female over 60 years old, BMI, HbA1c, DAS28-ESR, HAQ, mTSS, MTX dose, oral steroid dose, bDMARD use, RF, ACPA, CRP, TRACP-5b, homocysteine, BAP, Uc-OC, and pentosidine. We constructed a full multivariate regression model that included the all candidate explanatory parameters. The reduced multivariate regression model was made from a full model with stepwise methods. To check the interaction with each explanatory variable, the variance inflation factor (VIF) of each parameter was evaluated: all the VIF values were < 5; there were no severe interactions between each parameter. A *p* value < 0.05 was considered significant.

## Results

### Demographic data

A total of 379 patients were recruited to the KURAMA cohort from April to December 2012. Of these, 50 declined BMD testing, which resulted in 329 patients participating in the baseline checkup. After elimination of 85 patients because of missing, at least, one of the background data, the baseline dataset included 244 participants with full set of data available. These participants were followed up in 2014, and 30 of them declined to participate; thus, 214 patients were included in the final analysis (Fig. 1). The average and range of the age, BMI, and HbA1c were 63.2 (32–85) years, 21.3 (12.3–30.0), and 5.65 (4.6–9.2) respectively. Women over 60 years old who are expected to be in menopause were 61.1% (*n* = 131) of the participants [27]. A total of 27 patients (13%) had diabetes. Women comprised of 91.1% of the participants. BP, VitD, and bDMARDs were used in 31.3%, 37.3%, and 31.3% of patients, respectively (Table 1). Parathyroid hormone, selective estrogen receptor modulator, and anti-receptor activator of nuclear factor-kappaB antibody were used only in 0.2%, 0.1%, and 0.8% of the patients at the baseline and were excluded from the further analyses. The mean observation period was 2.3 (1.2–3.5) years. The baseline demographic data were not significantly different between those who were included in the final analysis and those who were excluded from the analysis (data not shown). The annual BMD changes in the three sites were 0.14 ± 2.70 in the lumbar spine, 0.46 ± 1.63 in the proximal hip, and 1.14 ± 1.85 in the forearm without significant differences between any of these, respectively. During the study period, 15 patients (7.0%) suffered with new fractures. Compared to the newly fractured patients with the non-fractured 199 patients, the BMD of the lumbar spine, the total hip, and the distal forearm showed significant difference (*p* = 0.04, 0.02, and 0.001, respectively); the fractured patients had significantly lower BMD at all of the three sites.

**Table 1:** Baseline demographics and data

Parameters	Average
Age ± SD (min max) (years)	63.2 ± 11.4 (32–85)
Female, *n* (%)	195 (91.1)
Female over 60 years old, *n* (%)	131 (61.1)
BMI ± SD (min max) (kg/m^2^)	21.3 ± 3.1 (12.3–30.0)
HbA1c (NGSP) (min max) (%)	5.65 ± 0.5 (4.6–9.2)
DAS-ESR ± SD (min max)	3.2 ± 1.1 (0.5–6.6)
HAQ ± SD (min max)	0.8 ± 0.7 (0–2.9)
TSS ± SD (min max)	122.6 ± 108.0 (0–443)
MTX dose ± SD (mg) (min max)	5.6 ± 3.9 (0–14)
Steroid dose ± SD (mg) (min max)	1.77 ± 2.62 (0–15)
bDMARD use, *n* (%)	67 (31.3)
Bisphosphonate use, *n* (%)	67(31.3)
Vitamin D use, *n* (%)	80(37.3)
Observation period (min max) (years)	2.3 (1.2–3.5)
Lumbar spine BMD ± SD (min max)	86.2 ± 18.3 (45.8–151.0)
Total hip-BMD ± SD (min max)	81.8 ± 14.3 (45.0–119.0)
Distal forearm-BMD ± SD (min max)	82.3 ± 22.0 (34.0–140.0)
The annual lumbar spine-BMD change ± SD (min max)	− 0.14 ± 2.70
The annual total hip-BMD change ± SD (min max)	− 0.46 ± 1.63
The annual distal forearm-BMD change ± SD (min max)	− 1.15 ± 1.85
RF ± SD (min max) (U/ml)	86.0 ± 129.4 (0–812.8)
ACPA ± SD (min max) (U/ml)	125.9 ± 113.5 (0–300)
CRP ± SD (min max) (mg/dl)	0.66 ± 1.32 (0–11.7)
TRACP-5b ± SD (min max) (mU/dl)	320.0 ± 149.0 (68–877)
Homocysteine ± SD (min max) (nmol/ml)	9.7 ± 3.5 (3.2–28.0)
Uc-OC ± SD (ng/mL) (min max)	4.8 ± 4.1 (0–23.3)
BAP ± SD (μg/L) (min max)	15.6 ± 6.4 (5.8–43.6)
Urinary pentosidine ± SD(pg/ml)(min max)	47.1 ± 25.9 (11.5–196.0)

*BMI* body mass index, *HbA1c NGSP* Hemoglobin A_1C_ National Glycohemoglobin Standardization Program, *DAS28-ESR* disease activity score in 28 joints-erythrocyte sedimentation rate, *HAQ* health assessment questionnaire, *TSS* total Sharp/van der Heijde score, *MTX* methotrexate, *bDMARDs* biological disease-modifying anti-rheumatic drugs, *BMD* bone mineral density (data was shown with young adult mean), *RF* rheumatoid factor, *ACPA* anti-cyclic citrullinated peptide antibody, *CRP* C-reactive protein, *TRACP-5b* tartrate-resistant acid phosphatase 5b, *Uc-OC* undercarboxylated osteocalcin, *BAP* bone-specific alkaline phosphatase

### Laboratory and bone metabolism biomarkers

The average values of ACPA, CRP, TRACP-5b, homocysteine, and pentosidine were 125.9 (0–300) U/ mL, 0.66 (0–11.7) mg/dL, 320.0 (68–877) mU/dL, 9.7 (3.2–28.0) ng/mL, and 47.1 (11.5–196.0) pg/mL, respectively (Table 1).

### Candidate risk factors of annual BMD change for each anatomical site

#### Lumbar spine

In univariate analysis, the annual BMD change in the lumbar spine was significantly associated with the use of steroid, BP or VitD, and homocysteine. Homocysteine remained as the significant, consistent predictor of annual BMD change in the lumbar spine in both multivariate regression models (Table 2).

**Table 2:** Analysis of annual BMD change in the lumbar spine

Parameter	Univariate analysis			Multivariable regression analysis (full model)		Multivariable regression analysis (reduced model)	
	*t*	95%CI	*p*	*t*	95%CI	*t*	95%CI
Female over 60 years old	−1.96	− 0.03~0.27	0.051	1.80	− 0.05~0.06		
BMI	− 0.29	− 0.14~0.10	0.77	0.10	− 0.11~0.12		
HbA1c (NGSP)	0.66	− 0.47~0.94	0.51	0.56	− 0.55~0.98		
DAS28-ESR	0.06	− 0.32~0.34	0.95	1.98*	0.001~0.73	− 0.01	− 0.38~0.37
HAQ	0.48	− 0.38~0.63	0.63	0.20	− 0.60~0.73		
TSS	− 0.26	− 0.66~0.51	0.31	2.15*	0.001~0.008	1.62	− 0.001~ − 0.01
MTX dose	− 1.10	− 0.14~0.04	0.95	− 0.37	− 0.12~0.08		
Steroid dose	2.08	0.01~0.28	0.04*	1.43	− 0.04~0.27	1.54	−0.03~0.26
bDMARD	0.83	− 0.73~0.57	0.41	− 0.60	− 0.60~0.32		
Bisphosphonate	2.41	0.15~1.44	0.02*	007	− 0.02~0.82	2.45*	0.10~0.95
Vitamin D	2.34	0.11~1.29	0.02*	0.87	− 0.27~0.58		
RF	− 0.60	− 0.004~0.002	0.55	0.48	− 0.003~0.005		
ACPA	− 0.84	− 0.005~0.002	0.40	− 1.52	− 0.007~0.001	−1.53	− 0.01~0.001
CRP	0.23	− 0.25~0.31	0.82	− 2.48*	− 0.95~ − 0.11	0.01	−0.31~0.32
TRACP-5b	− 0.86	− 0.003~0.001	0.39	− 1.66	− 0.01~0.001		
Homocysteine	− 1.97	− 0.21~ − 0.0001	0.049*	− 2.55*	− 0.30~ − 0.04	− 2.38*	−0.26~ − 0.02
Uc-OC	− 0.96	− 0.13~0.05	0.34	0.45	− 0.09~0.03		
BAP	− 0.24	− 0.06~0.05	0.81	0.95	− 0.04~0.12		
Urine pentosidine	1.59	− 0.003~0.03	0.11	1.63	− 0.002~0.03	1.45	−0.004~0.03

*BMD* bone mineral density, *BMI* body mass index, *DAS28-ESR* disease activity score in 28 joints-erythrocyte sedimentation rate, *HAQ* health assessment questionnaire, *TSS* total Sharp/van der Heijde score, *MTX* methotrexate, *bDMARDs* biological disease-modifying anti-rheumatic drugs, *RF* rheumatoid factor, *ACPA* anti-cyclic citrullinated peptide antibody, *CRP* C-reactive protein, *TRACP-5b* tartrate-resistant acid phosphatase 5b, *Uc-OC* undercarboxylated osteocalcin, *BAP* bone-specific alkaline phosphatase

*p ≥ 0.05

#### Proximal femur

In univariate analyses, the annual BMD change in the proximal femur was associated with DAS28-ESR, the use of BP or VitD, CRP, and ACPA. In the two multivariate regression models, BP, VitD, and ACPA remained the significant predictors of annual BMD change in the proximal femur (Table 3).

**Table 3:** Analysis of annual BMD change in the proximal femur

Parameter	Univariate analysis			Multivariable regression analysis (full model)		Multivariable regression analysis (reduced model)	
	*t*	95%CI	*p*	*t*	95%CI	*t*	95%CI
Female over 60 years old	−0.30	− 0.52~0.38	0.76	− 0.12	− 0.27~0.24		
BMI	−0.85	− 0.10~0.04	0.40	0.26	− 0.07~0.09		
HbA1c (NGSP)	−0.90	− 0.67~0.25	0.36	−1.04	− 0.76~0.24	0.12	− 0.80~0.09
DAS28-ESR	−1.98	− 0.40~ − 0.001	0.049*	0.06	− 0.25~0.26		
HAQ	0.48	− 0.38~0.63	0.63	− 0.81	− 0.56~0.24	−1.65	− 0.56~0.05
TSS	−0.26	− 0.66~0.51	0.31	− 0.40	− 0.003~0.002		
MTX dose	−0.12	− 0.06~0.05	0.90	− 0.52	− 0.08~0.05		
Steroid dose	0.52	− 0.06~0.11	0.60	0.05	− 0.09~0.10		
bDMARD	1.89	− 0.02~0.92	0.06	0.35	− 0.15~0.42		
Bisphosphonate	3.08	0.26~1.20	0.002*	3.53*	0.20~0.72	3.19*	0.15~0.62
Vitamin D	2.58	0.13~1.04	0.01*	2.72*	0.10~0.59	2.99*	0.12~0.58
RF	−0.60	− 0.004~0.002	0.55	1.07	− 0.001~0004		
ACPA	−2.09	− 0.004~ − 0.0001	0.04*	− 2.34*	− 0.01~ − 0.001	− 2.42*	− 0.004~ − 0.0004
CRP	−4.03	− 0.49~ − 0.17	< 0.001*	− 0.71	− 0.36~0.17		
TRACP-5b	−1.89	− 0.003~0.001	0.06	−1.00	− 0.003~0.001		
Homocysteine	−1.81	− 0.12~ − 0.005	0.07	− 018	− 0.09~0.07		
Uc-OC	0.66	− 0.06~0.04	0.65	0.68	− 0.04~0.09		
BAP	−1.20	− 0.05~0.01	0.23	0.68	− 0.03~0.06		
Urine pentosidine	−0.71	− 0.01~0.006	0.47	− 0.08	− 0.01~0.01		

*BMD* bone mineral density, *BMI* body mass index, *DAS28-ESR* disease activity score in 28 joints-erythrocyte sedimentation rate, *HAQ* health assessment questionnaire, *TSS* total Sharp/van der Heijde score, *MTX* methotrexate, *bDMARDs* biological disease-modifiying anti-rheumatic drugs, *RF* rheumatoid factor, *ACPA* anti-cyclic citrullinated peptide antibody, *CRP* C-reactive protein, *TRACP-5b* tartrate-resistant acid phosphatase 5b, *Uc-OC* undercarboxylated osteocalcin, *BAP* bone-specific alkaline phosphatase

*p ≥ 0.05

#### Distal forearm

In univariate analysis, the annual BMD change in the distal forearm was significantly associated with the dosage of MTX, the use of BP or VitD, and TRACP-5b. In the two multivariate regression models, the use of BP or VitD and TRACP-5b remained the significant predictors of annual BMD change in the distal forearm (Table 4). The results of the multivariate analyses for these three anatomical sites are summarized in Table 5.

**Table 4:** Analysis of annual BMD change in the distal forearm

Parameter	Univariate analysis			Multivariable regression analysis (full model)		Multivariable regression analysis (reduced model)	
	*t*	95%CI	*p*	*t*	95%CI	*t*	95%CI
Female over 60 years old	0.91	− 0.28~0.75	0.37	1.17	− 0.11~0.45		
BMI	− 0.46	− 0.10~0.06	0.64	0.87	− 0.05~0.13		
HbA1c (NGSP)	− 0.48	− 0.65~0.40	0.63	− 0.43	− 0.68~0.44		
DAS28-ESR	− 1.47	− 0.40~0.06	0.14	0.98	− 0.28~0.29		
HAQ	0.49	− 0.26~0.43	0.63	− 1.05	− 0.69~0.21		
TSS	1.39	− 0.001~0.004	0.31	1.43	− 0.001~0.004	1.64	− 0.0003~0.004
MTX dose	− 2.23	− 0.13~ − 0.01	0.03*	− 0.64	− 0.10~0.05		
Steroid dose	1.56	− 0.02~0.17	0.12	0.21	− 0.10~0.12		
bDMARD	1.34	− 0.17~0.90	0.18	0.05	− 0.31~0.32		
Bisphosphonate	2.45	0.13~1.20	0.02*	2.04*	0.01~0.59	2.06*	0.01~0.55
Vitamin D	2.26	0.07~1.10	0.03*	2.63*	0.10~0.64	2.07*	0.01~0.52
RF	0.28	− 0.002~0.002	0.79	− 0.39	− 0.001~0.004		
ACPA	0.93	− 0.001~0.003	0.35	1.17	− 0.001~0.004		
CRP	− 0.51	− 0.24~0.14	0.61	− 0.83	− 0.42~0.17		
TRACP-5b	− 2.54	− 0.004~ − 0.005	0.01*	− 3.18*	− 0.01~ − 0.001	−3.27*	− 0.005~ − 0.001
Homocysteine	0.71	− 0.05~0.10	0.48	− 0.17	− 0.10~0.08		
Uc-OC	− 0.83	− 0.09~0.04	0.40	0.05	− 0.07~0.08		
BAP	− 0.41	− 0.05~0.03	0.68	1.51	− 0.01~0.10	1.80	− 0.004~0.10
Urine pentosidine	0.47	− 0.01~0.01	0.64	0.96	− 0.005~0.02		

*BMD* bone mineral density, *BMI* body mass index, *DAS28-ESR* disease activity score in 28 joints-erythrocyte sedimentation rate, *HAQ* health assessment questionnaire, *TSS* total Sharp/van der Heijde score, *MTX* methotrexate, *bDMARDs* biological disease-modifying anti-rheumatic drugs, *RF* rheumatoid factor, *ACPA* anti-cyclic citrullinated peptide antibody, *CRP* C-reactive protein, *TRACP-5b* tartrate-resistant acid phosphatase 5b, *Uc-OC* undercarboxylated osteocalcin, *BAP* bone-specific alkaline phosphatase

*p ≥ 0.05

**Table 5:** Summary of multivariable regression analysis

Annual BMD change rate	Lumbar	Proximal femur	Distal forearm
Bisphosphonate		↑↑↑	↑
Vitamin D		↑↑	↑
ACPA		↓↓	
TRACP-5b			**↓↓↓**
Homocysteine	**↓**		

↑↑↑, 3 ≦ *t* < 3.5 and *p* < 0.05

↑↑, 2.5 ≦ *t* < 3.0 and *p* < 0.05

↑, 2 ≦ t < 2.5 and *p* < 0.05

↓, − 2.5 ≦ *t*< − 2 and *p* < 0.05

↓↓, − 3 ≦ *t* < − 2.5 and *p* < 0.05

↓↓↓, − 3.5 ≦ *t* < − 3 and *p* < 0.05

*BMD* bone mineral density, *ACPA* anti-cyclic citrullinated peptide antibody, *TRACP-5b* tartrate-resistant acid phosphatase 5b

## Discussion

This longitudinal cohort study identified distinct biomarkers for BMD changes at each of three anatomical sites. We demonstrated that the predictors of BMD in the lumbar spine were serum homocysteine, whereas the predictors of BMD were ACPA in the proximal femur and serum TRACP-5b in the distal forearm, respectively, along with osteoporosis drugs BP and VitD (summarized in Table 5). To the best of our knowledge, this is the first prospective, longitudinal study showing the distinct differences of biomarkers in different anatomical sites predicting the changes of BMD in patients with RA.

The rationale for and the mechanisms underlying these differences in bone biomarkers at each anatomical site has been little studied. Souza-Faloni et al. demonstrated that osteoclasts from different bone sites appear to differ in many respects. They reported that bone marrow cells from different places in the skeleton have different dynamics of osteoclast genesis and that these differences are mainly related to differences in the cellular conformation of the site-specific bone marrow [28]. Alternatively, Fehérvári demonstrated a body site-specific link between the severity of atherosclerosis and osteoporosis in patients with peripheral artery disease [29]. Further, de Carvalho et al. argued that long-lasting kidney disease, which is another disease causing secondary osteoporosis, is associated with poor BMD at the hip but not at the spine [30]. Therefore, the differences in risk factors and predictive biomarkers that we observed are reasonable from the perspective of bone biology.

This study identified serum homocysteine as a predictive biomarker for the change in BMD in the lumbar spine. A previous animal study showed that high homocysteine levels induce bone loss [31], while another report suggested that high serum homocysteine might influence bone mineral density, bone turnover, bone blood flow, and collagen cross-linking [32]. Homocysteine is known to be associated with inflammatory processes [33], but, in this study, CRP was not a significant predictor of homocysteine level, suggesting that the influence of systemic inflammation may not be directly associated with the homocysteine level. Indeed, Bahtiri et al. reported that serum homocysteine levels were inversely related to lumbar spine BMD and femur neck BMD in women with osteoporosis [34]. The reason why homocysteine was identified as a predictive biomarker of BMD change only in the lumbar spine remains to be investigated, but it is possible that homocysteine tends to accumulate in the spine, which has less cortical bone and more cancellous bone than the other two sites [35].

Another interesting finding of this study was that ACPA was a significant predictor of BMD change in the proximal femur. ACPA is known as a risk factor not only of joint destruction but of bone loss in RA patients [36]. Moreover, a few reports showed significant associations between ACPA and atherosclerosis or ischemic heart disease in RA patients [37]. Taken together, these findings suggest that the proximal femur may be particularly affected by vascular conditions or may be strongly shared in common pathophysiology between bone and vascular metabolism, but these notions should be further investigated.

In contrast, the predictive biomarkers for BMD in the distal forearm remain largely unknown. This study demonstrated that RA patients with higher TRACP-5b tended to lose BMD in the distal forearm in the 2-year period. It is well known that TRACP-5b is predominantly expressed in bone by osteoclasts [38]. Janckila et al. reported that the mean level of TRACP-5b protein was elevated in RA patients compared with healthy controls and other disease groups [39]. They suggested that TRACP-5b activity is a marker of osteoclast number and local or systemic bone destruction, which suggests the hypothesis that osteoclast activity induced by local and/or systemic inflammation might strongly influence bone metabolism, particularly in the distal forearm of RA patients.

One of the noteworthy results of this study is that the biomarkers identified were more potent than other known predictors of BMD changes such as age plus menopause, DM, and the use of steroids, possibly because this study was conducted relatively in a short term, and because the participants were predominantly women with RA. Nonetheless, in the current clinical setting where patients are treated using a T2T strategy, it can be argued that the biomarkers would be a powerful tool to predict the changes in BMD of patients with RA.

One question that remains is how differently each osteoporosis drug affects BMD at each anatomical site. Few studies have investigated the differences between the effects of different osteoporosis drugs on different bones. This study revealed that any drugs did not sufficiently affect the change of BMD in the lumber spine, whereas both BP and VitD affected that in the proximal femur and in the distal forearm. Moreover, the potency of BP may differ among the three sites because *t* value of multiple regression analysis in the reduced model was higher in the proximal femur (*t* = 3.19) than that in the distal forearm (*t* = 2.06). Golub et al. previously demonstrated that skeletal biodistribution of bisphosphonate is anatomic site-dependent in a rat model [40]. While the earlier studies for primary osteoporosis show that BP increases BMD in lumbar spine but does not significantly increase BMD in forearm [41], another previous report demonstrated that bone loss and bone turnover at the distal radius were significantly faster in RA patients than the general population [42]. In addition, the hip and the distal forearm are distinguished from the lumbar spine with more cortical bones than the lumbar spine and are mechanically related to the joints: the bone turnover of the two sites may be enhanced by periarticular osteoporosis compared with lumbar spine [43, 44]. Therefore, the difference of drug effect might reflect differences of drug distribution, the ratios of the cortical and the cancellous bone, and mechanical burdens at each anatomical site. Additional investigation would identify differences in the predictive biomarkers for each anatomical site and may reveal differences in the effects of different osteoporosis drugs.

This study has several limitations. First, the number of participants may not be large enough to identify more significant biomarkers for each anatomical site. Indeed, this study did not include all of biomarkers available due to practical reasons, and studies with more samples and biomarkers may reveal a different set of biomarkers for these anatomical sites. However, the biomarkers distinguished in this study remained significant in each analysis and should be considered reliable for prediction of BMD changes. Second, as reported elsewhere, a decrease in BMD may not necessarily indicate actual bone fragility. However, there is consensus that BMD determined by DEA is one of the most reliable and usable surrogate markers for assessing the risk of fracture in osteoporosis patients. Third, this was a single-center study, which could have led to some selection bias. Fourth, the follow-up period of this study was relatively short. However, the longer the patients are followed, the more confounding factors, such as changes in medication, must be taken into count, which in turn requires greater sample numbers and more complex statistical analyses. Fifth, this study did not include newer osteoporosis drugs such as parathyroid hormone, selective estrogen receptor modulator, and anti-receptor activator of nuclear factor-kappaB antibody for analyses. The influence of these drugs on selecting biomarkers remains to be investigated. Lastly, although we investigated six bone metabolism biomarkers plus general and RA-related biomarkers such as ACPA, other reported biomarkers may be more potent than those assessed in the current study. However, we selected established biomarkers that were representative of different aspects of bone metabolism, inflammation, and serological aspects of RA, which would have covered crucial aspects of pathophysiology of osteoporosis and RA. Which biomarkers are best in a particular clinical setting should be investigated in another study.

Despite the above limitations, no previous longitudinal study has evaluated the relationship of six types of bone metabolism markers and RA-specific parameters with changes in BMD at three body sites in a large RA cohort. In addition, we applied well-known influential factors such as menopause, DM, BMI, steroids, osteoporotic drugs, and bDMARDs as explanatory parameters in the full multiple regression model, which should lead to reliable results. Indeed, the results of the two regression models extracted the similar significant markers in each analysis. Therefore, our results suggest that the biomarkers identified in this study should be considered useful for BMD management in patients with RA treated by the current T2T strategy and osteoporosis drugs.

## Conclusions

This prospective longitudinal study identified distinct predictive biomarkers of BMD in the lumbar spine, proximal femur, and distal forearm in patients with RA. Multivariate analyses revealed that the significant predictors of BMD in the lumbar spine, proximal femur, and distal forearm were homocysteine, ACPA, and TRACP-5b, respectively, along with osteoporosis drugs BP and VitD. Practitioners and patients with RA should treat each anatomical site differently to prevent fractures and manage osteoporosis.

## Data Availability

All of the data supporting the findings are available upon request.

## Abbreviations

ACPA: Anti-cyclic citrullinated peptide antibody
BAP: Bone-specific alkaline phosphatase
bDMARDs: Biological disease-modifying anti-rheumatic-drugs
BMD: Bone mineral density
BMI: Body mass index
CRP: C-reactive protein
DAS28-ESR: Disease activity score in 28 joints-erythrocyte sedimentation rate
HAQ: Health Assessment Questionnaire
HbA1c NGSP: Hemoglobin A_1C_ National Glycohemoglobin Standardization Program
MTX: Methotrexate
RF: Rheumatoid factor
TRACP-5b: Tartrate-resistant acid phosphatase 5b
TSS: Total Sharp/van der Heijde score
Uc-OC: Undercarboxylated osteocalcin

## References

[1] Sattui SE, Saag KG (2014). Fracture mortality: associations with epidemiology and osteoporosis treatment. Nat Rev Endocrinol.

[2] Hauger AV (2018). Osteoporosis and osteopenia in the distal forearm predict all-cause mortality independent of grip strength: 22-year follow-up in the population-based Tromsø study.

[3] Kim D, Cho SK, Choi CB (2016). Incidence and risk factors of fractures in patients with rheumatoid arthritis: an Asian prospective cohort study. Rheumatol Int.

[4] Mazzucchelli R, Pérez Fernandez E, Crespí-Villarías N (2018). Trends in hip fracture in patients with rheumatoid arthritis: results from the Spanish National Inpatient Registry over a 17-year period (1999-2015). TREND-AR study. RMD Open.

[5] Jin S, Hsieh E, Peng L (2018). Incidence of fractures among patients with rheumatoid arthritis: a systematic review and meta-analysis. Osteoporos Int.

[6] Wilson J., Bonner T. J., Head M., Fordham J., Brealey S., Rangan A. (2009). Variation in bone mineral density by anatomical site in patients with proximal humeral fractures. The Journal of Bone and Joint Surgery. British volume.

[7] Ishida O, Furuya T, Inoue E (2015). Risk factors for established vertebral fractures in Japanese patients with rheumatoid arthritis: results from a large prospective observational cohort study. Mod Rheumatol.

[8] Furuya T, Inoue E, Hosoi T (2013). Risk factors associated with the occurrence of hip fracture in Japanese patients with rheumatoid arthritis: a prospective observational cohort study. Osteoporos Int.

[9] Ochi K, Go Y, Furuya T (2014). Risk factors associated with the occurrence of distal radius fractures in Japanese patients with rheumatoid arthritis: a prospective observational cohort study. Clin Rheumatol.

[10] Ochi K, Furuya T, Ikari K, et al. Sites, frequencies, and causes of self-reported fractures in 9,720 rheumatoid arthritis patients: a large prospective observational cohort study in Japan. Arch Osteoporos. 2013;8. 10.1007/s11657-013-0130-7. 10.1007/s11657-013-0130-7 23526031

[11] Vasikaran S, Eastell R, Bruyère O (2011). Markers of bone turnover for the prediction of fracture risk and monitoring of osteoporosis treatment: a need for international reference standards. Osteoporos Int.

[12] Wysham KD, Shoback DM, Imboden JB, Katz PP (2018). Association of high anti–cyclic citrullinated peptide seropositivity and lean mass index with low bone mineral density in rheumatoid arthritis. Arthritis Care Res.

[13] Haugeberg G, Uhlig T, Falch JA (2000). Bone mineral density and frequency of osteoporosis in female patients with rheumatoid arthritis: results from 394 patients in the Oslo County rheumatoid arthritis register. Arthritis Rheum.

[14] Kalvesten J, Haugeberg G, Elden A (2008). Adalimumab therapy reduces hand bone loss in early rheumatoid arthritis: explorative analyses from the PREMIER study. Ann Rheum Dis.

[15] Dubrovsky AM, Lim MJ, Lane NE (2018). Osteoporosis in rheumatic diseases: anti-rheumatic drugs and the skeleton. Calcif Tissue Int.

[16] Iwata T., Ito H., Furu M., Hashimoto M., Fujii T., Ishikawa M., Yamakawa N., Terao C., Azukizawa M., Hamamoto Y., Mimori T., Akiyama H., Matsuda S. (2015). Periarticular osteoporosis of the forearm correlated with joint destruction and functional impairment in patients with rheumatoid arthritis. Osteoporosis International.

[17] Nakagami Y, Sugihara G, Takei N, et al. Effect of physical state on pain mediated through emotional health in rheumatoid arthritis. Arthritis Care Res (Hoboken) 0–2. 2018. 10.1002/acr.23779. 10.1002/acr.23779 30295427

[18] Sharp John T., Strand Vibeke, Leung Hoi, Hurley Frank, Loew-Friedrich Iris (2000). Treatment with leflunomide slows radiographic progression of rheumatoid arthritis: Results from three randomized controlled trials of leflunomide in patients with active rheumatoid arthritis. Arthritis & Rheumatism.

[19] van der Heijde D (2004). Long term evaluation of radiographic disease progression in a subset of patients with rheumatoid arthritis treated with leflunomide beyond 2 years. Annals of the Rheumatic Diseases.

[20] Lau E. M. C., Leung P. C., Kwok T., Woo J., Lynn H., Orwoll E., Cummings S., Cauley J. (2005). The determinants of bone mineral density in Chinese men—results from Mr. Os (Hong Kong), the first cohort study on osteoporosis in Asian men. Osteoporosis International.

[21] Nakamura K., Tanaka Y., Saitou K., Nashimoto M., Yamamoto M. (2000). Age and Sex Differences in the Bone Mineral Density of the Distal Forearm Based on Health Check-up Data of 6343 Japanese. Osteoporosis International.

[22] Melton LJ, Looker AC, Shepherd JA (2005). Osteoporosis assessment by whole body region vs. site-specific DXA. Osteoporos Int.

[23] Ohnaru K, Sone T, Tanaka K (2013). Hip structural analysis: a comparison of DXA with CT in postmenopausal Japanese women. Springerplus.

[24] Iwamoto J, Takeda T, Ichimura S (2002). Forearm bone mineral density in postmenopausal women with rheumatoid arthritis. Calcif Tissue Int.

[25] Lloyd JT, Alley DE, Hochberg MC (2016). Changes in bone mineral density over time by body mass index in the health ABC study. Osteoporos Int.

[26] Clowes JA, Hannon RA, Yap TS (2002). Effect of feeding on bone turnover markers and its impact on biological variability of measurements. Bone.

[27] Hill K (1996). The demography of menopause. Maturitas.

[28] de Souza Faloni Ana Paula, Schoenmaker Ton, Azari Azin, Katchburian Eduardo, Cerri Paulo S., de Vries Teun J., Everts Vincent (2010). Jaw and Long Bone Marrows Have a Different Osteoclastogenic Potential. Calcified Tissue International.

[29] Fehérvári M, Sarkadi H, Krepuska M (2013). Bone mineral density is associated with site-specific atherosclerosis in patients with severe peripheral artery disease. Calcif Tissue Int.

[30] Bezerra de Carvalho K. S., Vasco R.F.V., Custodio M.R., Jorgetti V., Moysés R.M.A., Elias R.M. (2019). Chronic kidney disease is associated with low BMD at the hip but not at the spine. Osteoporosis International.

[31] Behera J, George AK, Voor MJ (2018). Hydrogen sulfide epigenetically mitigates bone loss through OPG/RANKL regulation during hyperhomocysteinemia in mice. Bone.

[32] Fratoni V, Brandi ML (2015). B vitamins, homocysteine and bone health. Nutrients.

[33] Peyrin-Biroulet L, Rodriguez-Guéant RM, Chamaillard M (2007). Vascular and cellular stress in inflammatory bowel disease: revisiting the role of homocysteine. Am J Gastroenterol.

[34] Bahtiri E, Bahtiri E, Islami H (2015). Relationship of homocysteine levels with lumbar spine and femur neck BMD in postmenopausal women relationship of homocysteine levels with lumbar spine and femur neck BMD in postmenopausal women. Acta Reumatol Port.

[35] Herrmann M, Garcia P, Tami A (2008). Hyperhomocysteinemia induces a tissue specific accumulation of homocysteine in bone by collagen binding and adversely affects bone. Bone.

[36] Ozer C, Guler H, Turhanoglu AD (2008). The relationship between anti-cyclic citrullinated peptide and bone mineral density and radiographic damage in patients with rheumatoid arthritis. Scand J Rheumatol.

[37] Gerli R, Bocci EB, Sherer Y (2007). Association of anti-cyclic citrullinated peptide antibodies with subclinical atherosclerosis in patients with rheumatoid arthritis. Ann Rheum Dis.

[38] Gradin P, Hollberg K, Cassady AI (2012). Transgenic overexpression of tartrate-resistant acid phosphatase is associated with induction of osteoblast gene expression and increased cortical bone mineral content and density. Cells Tissues Organs.

[39] Janckila AJ, Neustadt DH, Yam LT (2008). Significance of serum TRACP in rheumatoid arthritis. J Bone Miner Res.

[40] Golub E, Qing L, Wen D (2010). Anatomic site variability in rat skeletal uptake and desorption of fluorescently labeled bisphosphonate. Oral Dis.

[41] Paggiosi MA, Peel N, Mccloskey E, Walsh JS (2014). Comparison of the effects of three oral bisphosphonate therapies on the peripheral skeleton in postmenopausal osteoporosis : the TRIO study. Osteoporos Int.

[42] Sambrook P N, Ansell B M, Foster S, Gumpel J M, Hesp R, Reeve J (1985). Bone turnover in early rheumatoid arthritis. 2. Longitudinal bone density studies. Annals of the Rheumatic Diseases.

[43] Alenfeld FE, Diessel E, Brezger M (2000). Detailed analyses of periarticular osteoporosis in rheumatoid arthritis. Osteoporos Int.

[44] Schett G, Gravallese E (2012). Bone erosion in rheumatoid arthritis: mechanisms, diagnosis and treatment. Nat Rev Rheumatol.

